# Combining empirical knowledge, in silico molecular docking and ADMET profiling to identify therapeutic phytochemicals from *Brucea antidysentrica* for acute myeloid leukemia

**DOI:** 10.1371/journal.pone.0270050

**Published:** 2022-07-27

**Authors:** Lemessa Etana Bultum, Gemechu Bekele Tolossa, Doheon Lee

**Affiliations:** 1 Department of Bio and Brain Engineering, Korea Advanced Institute of Science and Technology (KAIST), Daejeon, South Korea; 2 Bio-Synergy Research Center, Daejeon, South Korea; 3 Department of Neuroscience, Washington University School of Medicine, St. Louis, Missouri, United States of America; Adama Science and Technology University, ETHIOPIA

## Abstract

Acute myeloid leukemia (AML) is one of the deadly cancers. Chemotherapy is the first-line treatment and the only curative intervention is stem cell transplantation which are intolerable for aged and comorbid patients. Therefore, finding complementary treatment is still an active research area. For this, empirical knowledge driven search for therapeutic agents have been carried out by long and arduous wet lab processes. Nonetheless, currently there is an accumulated bioinformatics data about natural products that enabled the use of efficient and cost effective in silico methods to find drug candidates. In this work, therefore, we set out to computationally investigate the phytochemicals from *Brucea antidysentrica* to identify therapeutic phytochemicals for AML. We performed in silico molecular docking of compounds against AML receptors IDH2, MCL1, FLT3 and BCL2. Phytochemicals were docked to AML receptors at the same site where small molecule drugs were bound and their binding affinities were examined. In addition, random compounds from PubChem were docked with AML targets and their docking score was compared with that of phytochemicals using statistical analysis. Then, non-covalent interactions between phytochemicals and receptors were identified and visualized using discovery studio and Protein-Ligand Interaction Profiler web tool (PLIP). From the statistical analysis, most of the phytochemicals exhibited significantly lower (p-value ≤ 0.05) binding energies compared with random compounds. Using cutoff binding energy of less than or equal to one standard deviation from the mean of the phytochemicals’ binding energies for each receptor, 12 phytochemicals showed considerable binding affinity. Especially, hydnocarpin (-8.9 kcal/mol) and yadanzioside P (-9.4 kcal/mol) exhibited lower binding energy than approved drugs AMG176 (-8.6 kcal/mol) and gilteritinib (-9.1 kcal/mol) to receptors MCL1 and FLT3 respectively, indicating their potential to be lead molecules. In addition, most of the phytochemicals possessed acceptable drug-likeness and absorption, distribution, metabolism, excretion, and toxicity (ADMET) properties. Based on the binding affinities as exhibited by the molecular docking studies supported by the statistical analysis, 12 phytochemicals from *Brucea antidysentrica* (1,11-dimethoxycanthin-6-one, 1-methoxycanthin-6-one, 2-methoxycanthin-6-one, beta-carboline-1-propionic acid, bruceanol A, bruceanol D, bruceanol F, bruceantarin, bruceantin, canthin-6-one, hydnocarpin, and yadanzioside P) can be considered as candidate compounds to prevent and manage AML. However, the phytochemicals should be further studied using *in vivo & in vitro* experiments on AML models. Therefore, this study concludes that combination of empirical knowledge, in silico molecular docking and ADMET profiling is useful to find natural product-based drug candidates. This technique can be applied to other natural products with known empirical efficacy.

## Background

Leukemia is a group of cancers affecting early blood-forming cells. Acute myeloid leukemia (AML) is an aggressive malignancy of myeloid blood cells, which annually affects more than a million people globally and results in more than 100,000 deaths [1, 2]. According to American Cancer Society’s estimates for leukemia in the US for 2020, AML is the deadliest subtype of leukemia constituting 48% of Leukemia related deaths [3]. The first-line therapy for AML is chemotherapy [4, 5] and the five-year survival rate is about 28% for all patients and overall survival rate of AML patients across the world is still dismal [6]. The result in older patients who are unable to receive intensive chemotherapy is very bad with a median survival of only 5 to 10 months [7]. Although there has been some increase in survival for younger patients through better management of therapy-related toxicities, stem cell transplantation and emergence of some targeted drugs, AML’s therapy has largely remained unchanged for over four decades [8] and is still an active topic of research. Recent advances in high-throughput technologies enabled the elucidation of genetics and pathophysiology leading to detection of recurrent molecular mutations [9] in AML. FLT3 (Fms-like Tyrosine Kinase 3) is a receptor tyrosine kinase involved in hematopoiesis and reported to be the most commonly mutated in AML patients [10, 11]. FLT3 inhibitors such as quizartinib and gilteritinib, are being used in combination with standard chemotherapy to treat AML patients [11]. MCL1 (myeloid cell leukemia-1) is a closely related to BCL2 (B-cell lymphoma 2) and both frequently overexpressed in acute myeloid leukemia and critical for the survival of AML cells and AML stem cells [12, 13]. Venetoclax (ABT-199) is a novel, orally bioavailable small-molecule inhibitor for selective targeting of BCL2 [14]. AMG 176 is a potent, selective, and orally bioavailable MCL1 inhibitor that induces a rapid apoptosis in models of hematologic malignancies. The synergistic combination of AMG 176 and venetoclax demonstrates strong activity in models of AML at tolerated doses, highlighting the promise of BH3-mimetic combinations in hematologic cancers [15]. IDH1 (Isocitrate dehydrogenase) and IDH2 are two enzymes located in the cytoplasm/peroxysomes and mitochondria, respectively and point mutations of these enzymes are found in several malignancies including AML [16]. IDH inhibitors have shown good clinical response in AML patients and enasidenib and ivosidenib are approved by Food and Drug Administration (FDA) in 2017 and 2018 for the treatment of adult relapsed or refractory (R/R) AML with IDH2 and IDH1 mutations, respectively [17].

Natural products have long been a rich source for drug discovery. In four decades of 1981 to 2019, more than 40% (up to 85% with some criteria) of approved small-molecule anticancer therapeutic agents were naturally inspired [18]. There has been a continuing search of natural product or natural product inspired molecules for novel anticancer agents. The starting point of many of such natural product derived drugs is an empirical knowledge of their therapeutic effects that passes through generations. Therefore, it would be beneficial to explore traditionally known antileukemic agents as potential sources for treatment and management of AML. One such potential source is *Brucea antidysentrica (B*. *antidysentrica)*, a plant used to treat cancer in Ethiopia [19]. Inspired by this traditional and empirical knowledge, studies over the decades have investigated *B*. *antidysentrica* and *B*. *javanica* (a closely related plant species used in Chinese traditional medicine as an anticancer) for their anticancer activities both as crude extracts and specific compounds [19–22]. Quassinoids from *B*. *antidysentrica* such as bruceantin, bruceantinol, bruceantinoside C, brusatol, and bruceantinol have been identified to have antineoplastic activities in various cancers such as leukemia, breast carcinoma, melanoma, myeloma, and colon cancer [23–28]. More specifically, spurred by discovery of bruceantin’s anti-leukemic effect in 1973 by [24], other compounds of *B*. *antidysentrica* and *B*. *javanica* such as bruceantinoside A, bruceantinoside B, bruceanol A, bruceanol B, brusatol, and Yadanzioside P were also reported to have antileukemic activities [29–32]. The compounds from *B*. *antidysentrica* have even arrived on Phase II clinical trials for their anticancer activities [28, 33]. To this date, compounds from the *B*. *antidysentrica* and *B*. *javanica* are being investigated for their anticancer activities [27, 34].

These previous studies on anticancer effect of *B*. *antidysentrica* have been performed by extraction, identification of the plant’s compounds and testing them on various cancer cell lines using the long and arduous wet lab processes. As it is known, extraction and testing of compounds from natural products is a complicated and expensive process [35]. For example, the whole process of conventional drug discovery from lead identification to drug development is estimated to be 15 years [36] and it may cost around 800 million US dollars [37]. Even when there is an empirical knowledge of a traditional medicine against specific disease, it is difficult to identify all compounds from the plants/medicinal materials and test them for their pharmacological activities. However, numerous compounds are extracted from different medicinal materials at different times for various purposes. This resulted in buildup of numerous databases of potential therapeutic phytochemicals constituting the medicinal materials. These compound libraries can be screened for potential drugs using computational methods such as virtual screening, which is time and cost effective. The traditional medicine inspired reverse pharmacology is considered faster, economical and safe alternative because their safety evidence is accumulated from ancestors, the availability could be secured and can be accessed from mother nature with relatively low cost. Molecular docking is one of the widely used computational method in which the disease associated target protein is docked with large libraries of compounds using computer algorithms and their non-covalent binding strength is characterized by scoring functions [38]. Although molecular docking is a relatively recent in the field of natural products study, it has been extensively used in structure-based virtual screening since the early 1980s [39]. It is initiated by identifying disease related targets and attempts to predict the best interaction mode between target and candidate compounds to form a stable complex [40]. It is achieved through two steps: first by prediction of ligand conformations as well and its position and orientation in the binding site of the protein (also called binding poses) and then evaluating the binding affinity of each poses using a scoring function [41].

Natural products, which are used to treat various kinds of diseases, are usually multi herb or at least multi-compound concoctions. Although this multi-target polypharmacological approach might be an important aspect of traditional medicines, it is beneficial to identify specific phytochemical(s) that are actually involved in interacting with the relevant receptors in order to make targeted treatments, identify their action mechanisms and test their multi-targeting attributes. Such in silico molecular docking studies have been utilized to this end in identifying specific natural products’ compounds involved in treating various diseases [42–46]. In this work, we have demonstrated that empirical knowledge combined with in silico molecular docking and ADMET profiling is an effective method to identify the anti-leukemic potential of so-far identified *B*. *antidysentrica* phytochemicals. Since AML is morphologically and genetically heterogenous disease, we approached our work from a multi-target multi-ligand perspective. There are multiple investigational and approved AML drugs, which target various receptors. We investigated how well *B*. *antidysentrica* compounds can bind to these various targets and compared the values to the binding energy of the modern drugs, which we believe can be used as a gold standard for their respective targets. We believe this method can reveal the compounds, among phytochemicals of the plant, that might be bioactive and have anticancer activities. To the best of our knowledge, there were no previous efforts to combine empirical knowledge, in silico molecular docking, ADMET profiling and statistical analysis to investigate the therapeutic effect of phytochemicals from *B*. *antidysentrica*.

## Materials and methods

**Fig 1:**
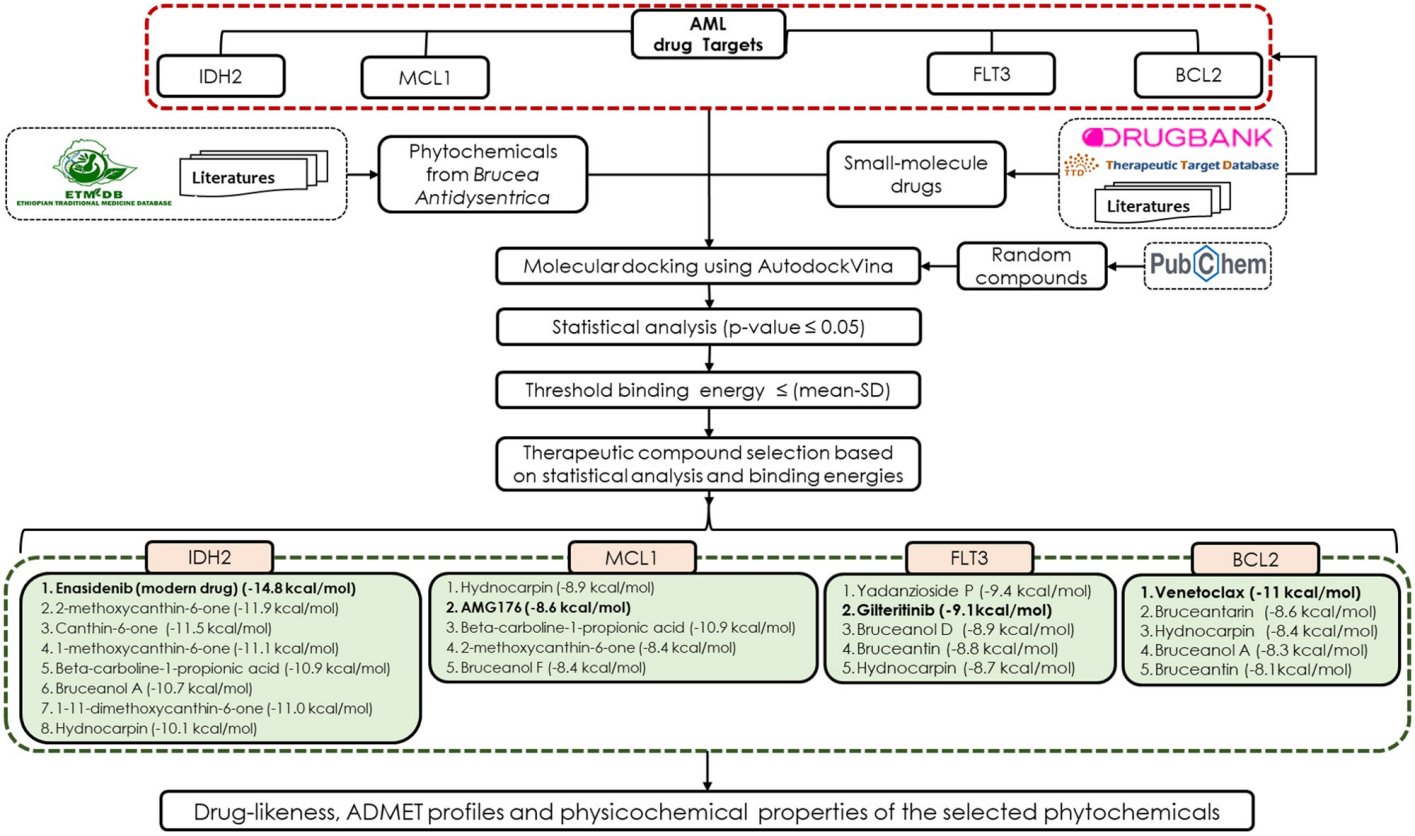
Schematic representation of the various processes used in the study. Briefly, AML targets and modern drugs (small molecule drugs) were obtained from drug bank, therapeutic target database (TTD) and/or literatures sources. *B*. *antidysentrica* phytochemicals were compiled from Ethiopian traditional medicine database (ETM-DB) and/or literatures. Then, the modern drugs, phytochemicals and random compounds (obtained from PubChem) were docked with AML targets. Binding energies of small molecule drugs and phytochemicals were evaluated against that of random compounds with statistical test. Finally, the candidate therapeutic phytochemicals were selected based on the statistical analysis and cutoff binding energies. In addition, the candidate compounds were further evaluated for drug-likeness, physicochemical and ADMET properties.

In this research, we used empirical knowledge combined with in silico molecular docking and DMET profiling to investigate the phytochemicals from traditional medicine plant *B*. *antidysentrica* for its potential therapeutic activity on AML. We identified the potential targets for the *B*. *antidysentrica* phytochemicals from the knowledge of the approved small molecules drugs which are currently being used to treat AML. Although there are multiple approved and investigational drugs and targets for AML, we selected four of the drugs and targets because their target-drug interaction complex has been elucidated using 3D crystallography and is publicly available. These drugs are enasidenib, AMG176, gilteritinib and venetoclax with their corresponding targets IDH2, MCL1, FLT3 and BCL2, respectively. The protein names of the targets, respectively, are Isocitrate dehydrogenase, Myeloid cell leukemia-1, Fms-like Tyrosine Kinase 3, and B-cell lymphoma 2. The 3 D Structure Data File (.SDF) file of the drugs are provided as supporting information (S1 File). The drug and target information were identified from the DrugBank [47], Therapeutic Target Database [48], and literature searches [15, 49, 50]. The 3D structures of the compounds were acquitted from PubChem Database [51], ChEMBL and/or ZINC database [52] and 3D structure of the targets were acquired from the Protein Data Bank (PDB) [53]. We selected x-ray crystallographic structures of AML targets with the small molecule drug complex. Then, in silico molecular docking was employed to predict the interactions between AML targets and compounds based on their poses and binding free energies, which are expressed as ligand–protein binding forces in kilocalories per mole (kcal/mol) [54]. We utilized AutoDock vina, which is one of the widely used bioinformatics tools for docking. The schematic representation of the overall process used in this research is shown in Fig 1.

### Acquisition and preparation of phytochemicals

**Fig 2:**
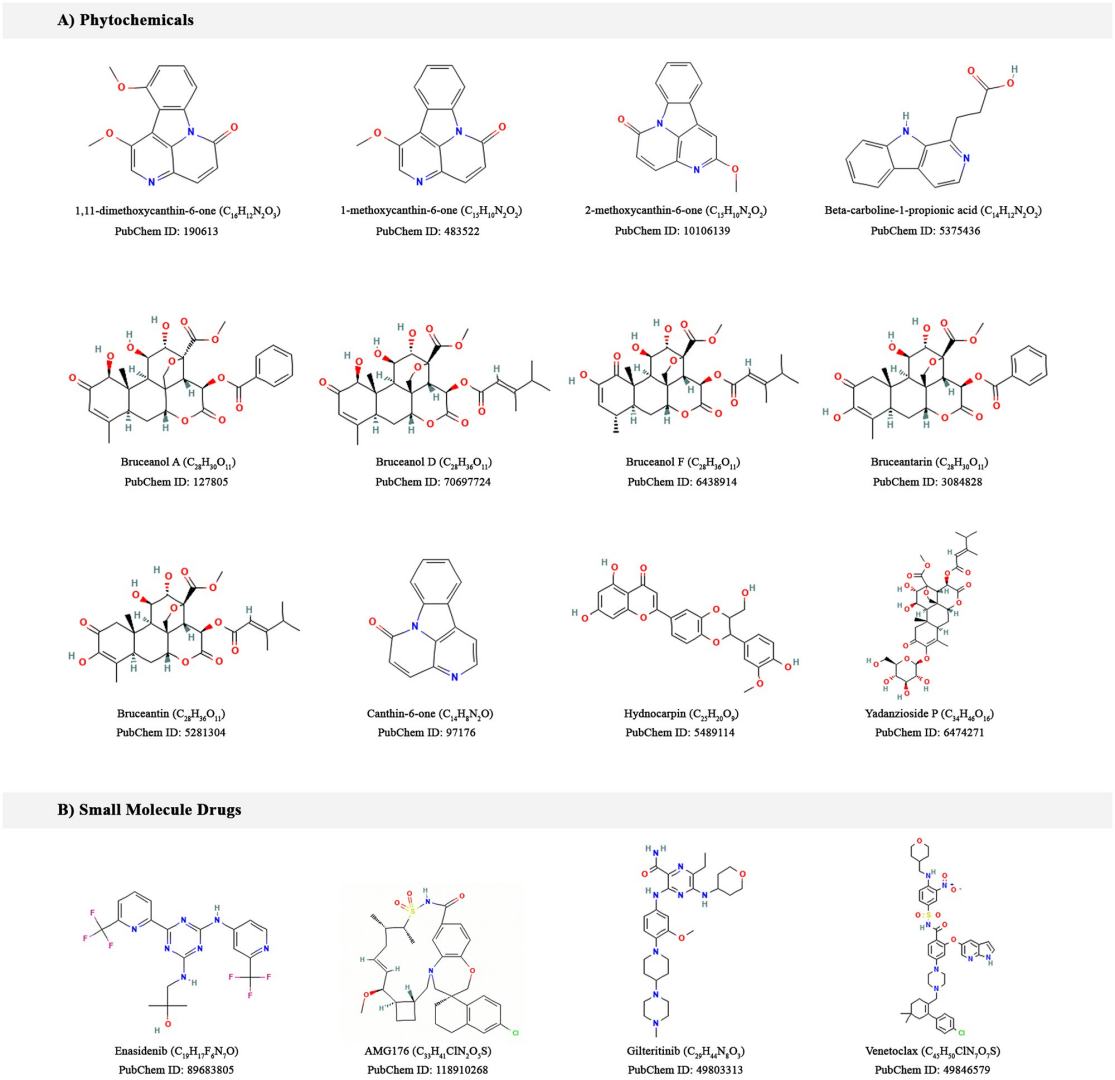
2D structures of the investigated compounds in this study. (A) Selected *B*. *antidysentrica* phytochemicals and (B) Small molecule drugs.

There has been long history of researches on anticancer activities of compounds from *B*. *antidysentrica*. Phytochemicals from this plant were reported to be effective against various cancer types such as leukemia, breast carcinoma, melanoma, pancreatic adenocarcinoma, and colon cancer [23–28]. From an extensive literature searches and Ethiopian traditional medicine database (ETM-DB) [55], we compiled compounds of *B*. *antidysentrica* (Fig 2A). The phytochemicals 3D SDF were downloaded the PubChem Database, ChEMBL and/or ZINC databases.

The SDF of the phytochemicals were converted to PDB format using open babel [56, 57], We then used MGL tools to convert the PDB files to PDBQT to prepare PDBQT for AutoDock Vina, a molecular docking software [58]. Docking algorithms require each atom to have a charge and an atom type that describes its properties. Therefore, both ligands and targets should be prepared properly to include these values. For each ligand, ADT automatically adds gasteiger charges, non-polar hydrogen atoms and detects rotatable bonds [59].

### Acquisition and preparation of molecular drug targets

Small molecule modern drugs and AML therapeutic targets were retrieved from Drug Bank database [60], Therapeutic Target Database (TTD) [61] and literature searches. The AML chemotherapeutic small molecule drugs used in this study were enasidenib, AMG176, gilteritinib and venetoclax with their target proteins: Isocitrate dehydrogenase (IDH2), Induced myeloid leukemia cell differentiation protein (MCL1), FLT3 and BCL2 respectively (Fig 2B). The crystallographic structures of the target protein and small molecule drug complexes were obtained from the Protein Data Bank (PDB) [62] with PDB IDs 5I96, 6O0F, 6JQR and 6O0K respectively.

The crystallographic structures PDB file contains protein, ligand, water oxygen atoms. Also, crystal structures normally lack polar hydrogen atoms which are required for appropriate treatment of electrostatics during docking [59]. For each protein, all the water molecules were removed, polar hydrogens and Kollman charges were added and the PDB files were converted to PDBQT to prepare for docking using ADT [58, 59].

### Molecular docking study

The 3D structure of the targets were small molecule drug-target complexes. We removed the small molecule drugs from the drug-target complexes using PyMol [63]. The positions of the small molecule drugs were considered as binding sites of the targets. The small molecule drugs were then re-docked to their corresponding targets and their binding scores were compared with that phytochemicals and random compounds. The binding energies of the small molecule drugs to their corresponding targets were much lower than the random compounds and most of the phytochemicals binding energy (see Results) indicating the rationality of our binding site identification method. Phytochemicals from *B*. *antidysentrica* and random compounds from PubChem were docked to AML receptors keeping all the docking parameters similar to the parameters of the small molecule drugs.

AutoDock Vina was used to do molecular docking in this work. During the docking procedure, both the protein and ligands were considered as rigid to minimize complexity that may arise from the large size of proteins and their multiple degrees of freedom [41]. All ligands (phytochemicals, small molecule drugs, and random compounds) were docked to individual receptors with grid coordinates and grid boxes of certain sizes for each receptor. The dimension of the grid box was set to a default value of 30 and the grid box centers were defined as the center of small molecule drug bound to the AML targets. The receptors and the grid center (x, y, z) used were as follows: 5I96 (-1.396, 15.123, -28.458), 6O0F (-14.521, 8.041, -18.915), 6JQR (-28.036, -10.299, -28.981), and 6O0K (-15.258, 2.212, -9.458) respectively. The exhaustiveness parameter that controls the extent of the search was set to 32. For each ligand, the pose with lowest energy of binding was retrieved and aligned with receptor structure for further analysis.

Eventually, compounds with computed docking energy of less than or equal to cutoff binding energy were selected as the candidate therapeutic phytochemicals. The cutoff binding energy for each AML target was set to the mean of the binding energies of all the phytochemicals minus their standard deviation (mean—SD in kcal/mol). The docking scores and the 2D and 3D pose views were generated for further analysis.

### Statistical analysis

In order to show that empirical knowledge combined with in silico molecular docking and ADMET profiling is one of the effective methods to identify natural product-based medicines and as an approach to validate our results, around 100 compounds from PubChem were docked with AML targets keeping all the docking parameters similar to for small molecule drugs and phytochemicals. Then a one-sample one-sided t-test was applied to measure the statistical significance (P ≤ 0.05) of the binding affinities of the small molecule drugs and *B*. *antidysentrica* phytochemicals compared to that of random compounds. We used python scripts to download the random compounds and to automate statistical analysis as well as to plot the docking scores for the compounds (small molecule drugs, phytochemicals, and random compounds) for each AML targets.

### Post docking analysis

The interactions between the compounds and the drug targets were examined in both 3D and 2D orientations. The docking poses were selected based on cutoff binding energies, which was set to less than or equal to one standard deviation from the mean of the phytochemicals’ binding energies for each receptor. Post docking analyses were carried out using PyMOL [63] and BIOVIA Discovery Studio [64]. PyMOL was used to visualize the docked protein and ligand complex binding pose and to convert the PDBQT format of AutoDock Vina output to PDB format. Then, the PDB format of the docked target protein and ligand complex were further visualized and analyzed using BIOVIA Discovery Studio, which showed the locations of binding sites and nature of interactions between the target protein and the docked ligand. Furthermore, non-covalently interacting residues of the target protein with the compounds were predicted using Protein-Ligand Interaction Profiler (PLIP) webserver [65].

### Drug-likeness properties, ADMET properties, and interactions with P-gp and CYP isoenzymes of the compounds

Evaluation of drug-likeness and ADMET properties, estimation for a compound to be substrate of P-gp or inhibitor of the most important CYP isoenzymes of the compounds were calculated using a website SwissADME [66], which is a free web tool to evaluate pharmacokinetics, drug-likeness and medicinal chemistry friendliness of small molecules. Toxicity of the phytochemicals and small molecule drugs were predicted using web interface of ProTox-II, which is user friendly and can predict potential toxicities associated with a chemical structure from user inputs of the name of the compound or the SMILES (Simplified Molecular-Input Line-Entry System) string of the compound [67]. In this work, we evaluated the acute toxicity of the compounds which are based on chemical similarities between compounds with known toxic effects and the presence of toxic fragments [68].

### Computation environment

All the locally installable software programs or scripts were implemented in an automatic fashion using Windows Command Prompt Commands and Python 3.8 on windows 10. All simulations were performed on a Windows 10 computer (Intel(R) Core (TM) i5-4690 CPU @ 3.50GHz, 3501 Mhz, 4 Core(s), and 4 Logical Processor(s)). The configuration of the RAM: 4 slots, 3 of them each having 8 GB RAM and 1 slot 4 GB RAM giving a combined 28 GB, DDR3 and speed of 1600MHz.

## Results and discussion

Acute myeloid leukemia (AML) is an aggressive malignancy of myeloid blood cells, which annually affects more than a million people globally and results in more than 100,000 deaths [1, 2]. The major intervention is stem cell transplantation which is unbearable for elderly and severe comorbid patients thereby necessitating for complementary and alternative therapies. Recent advances in high-throughput technologies enabled the elucidation of genetics and pathophysiology leading to identification therapeutic targets [9]. To this end, IDH2, MCL1, FLT3 and BCL2 are identified as drug targets for treatment of AML patients [10, 12, 13, 16].

In this research, we used information on the identified drug targets and small molecule drugs for treatment of AML to evaluate the therapeutic potential of phytochemicals from *B*. *antidysentrica* targeting AML receptors. The X-ray crystallographic structures of the AML receptors (IDH2, MCL1, FLT3 and BCL2) used in this research was downloaded from the PDB with PDB IDs 5I96, 6O0F, 6JQR and 6O0K respectively. Hence, we proposed to identify potential inhibitors of AML therapeutic target proteins by combining traditional empirical knowledge and in silico molecular docking. AutoDock Vina was reported as the best software to implement docking giving rise to poses that bind best deep inside the 5 Å of the binding pocket [69]. The docking process returned top ten ranked docking poses for each compound. In each case, we selected the topmost docking poses with Root Mean Square Deviation (RMSD) values of 0 Å.

From the 38 phytochemicals identified from *B*. *antidysentrica*, 30 of them have 3D structures in PubChem, ChEMBL and/or ZINC databases. The 3 D SDF file of these phytochemicals are provided as supporting information (S2 File). The remaining 8 compounds either lack 3D structures or their conformer generation were rejected. 3D representations for bruceanic acids B, C, and D are not available. For bruceanic acid A, yadanziosides G and M, bruceantinosides B and C, conformer generation were disallowed because of too many undefined stereo centers. In silico molecular docking was performed for phytochemicals with 3D structures.

**Fig 3:**
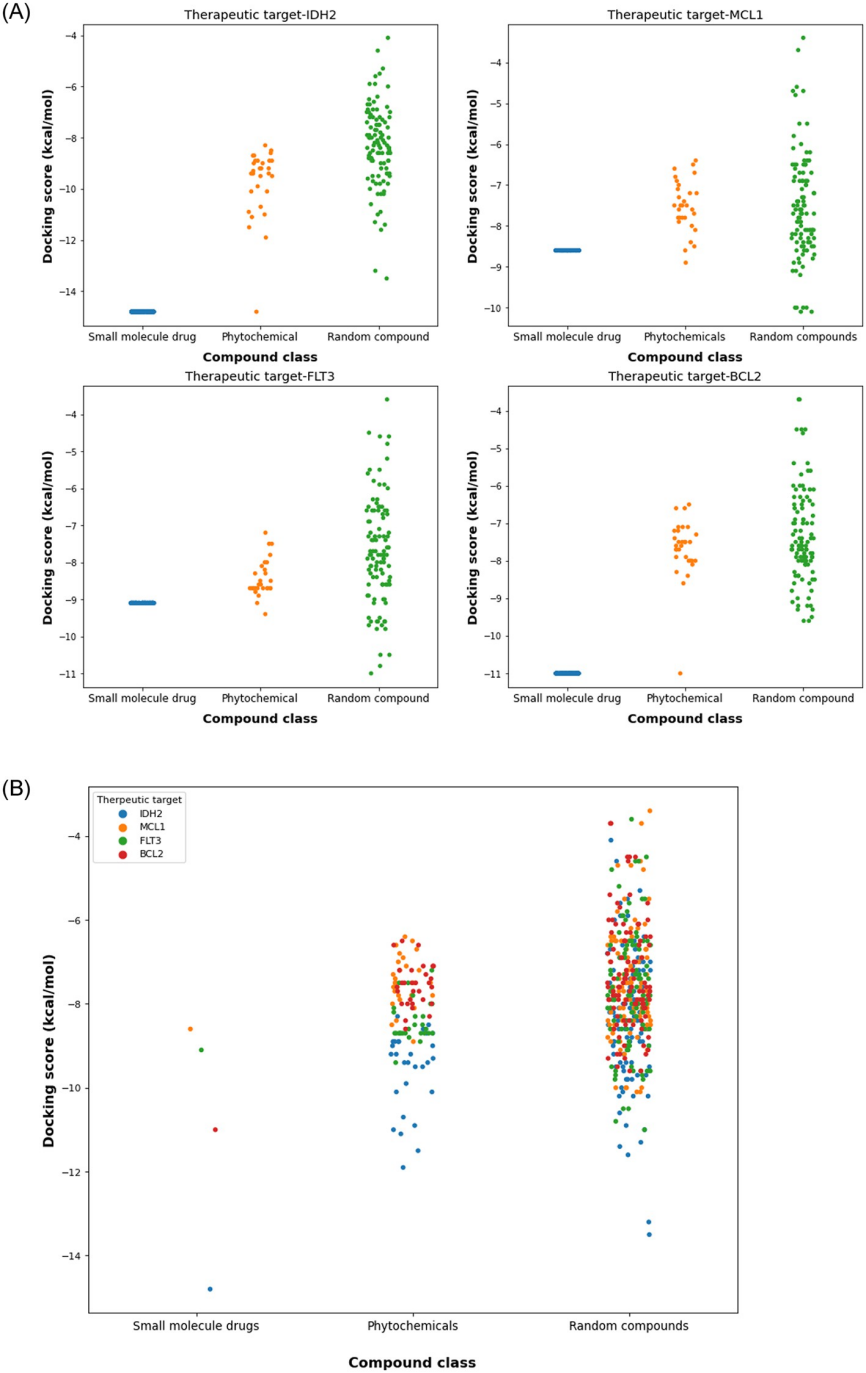
Scatter plots of the docking scores of compound-target interactions for each AML therapeutic targets. (A) compound—target docking score visualization for each AML targets, (B) compound—target docking scores combined for all the four AML targets.

The statistical analysis for testing the significance of the docking scores of *B*. *antidysentrica* phytochemicals compared to random compounds showed that most of the phytochemicals have significant lower binding affinities (p-value ≤ 0.05). The 3 D SDF file of random compounds used are provided as supporting information (S3 File). In addition to 3D structures, we provided 2D structures of all the investigated compounds as supporting information for reference (S4 and S5 Files). The binding energies of the of small molecule drugs, phytochemicals and random compounds are visualized in Fig 3A and 3B as a scatter plot indicating that the small molecule drugs and phytochemicals have lower binding energies than random compounds. The docking scores of the all investigated phytochemicals and the p-values are available in supporting information (S6 File). This result is a step toward validating the traditionally claimed empirical knowledge of the therapeutic potential of *B*. *antidysentrica* and its compounds against AML. Furthermore, based on the chosen cutoff energy (mean-SD), 12 phytochemicals showed substantial binding affinity compared to the standard approved drugs. Especially, hydnocarpin and yadanzioside P exhibited better binding affinity compared to the approved drugs which targets MCL1 and FLT3 receptors, respectively. Hydnocarpin has lower binding energy (-8.9 kcal/mol) with MCL1 receptor compared to the small molecule drug AMG176 (-8.6 kcal/mol) on the same receptor. Similarly, yadanzioside P has lower binding energy (-9.4 kcal/mol) to FLT3 receptor compared with the small molecule modern drug gilteritinib (-9.1 kcal/mol). Furthermore, phytochemicals bruceanol D (-8.9 kcal/mol), bruceantin (-8.8 kcal/mol), hydnocarpin (-8.7 kcal/mol) also have closely similar binding affinities to FLT3 receptor with approved drug gilteritinib. These results demonstrated that numerous phytochemicals from *B*. *antidysentrica* has considerable binding affinities with AML targets signifying their therapeutic potential for treatment and management of AML.

**Fig 4:**
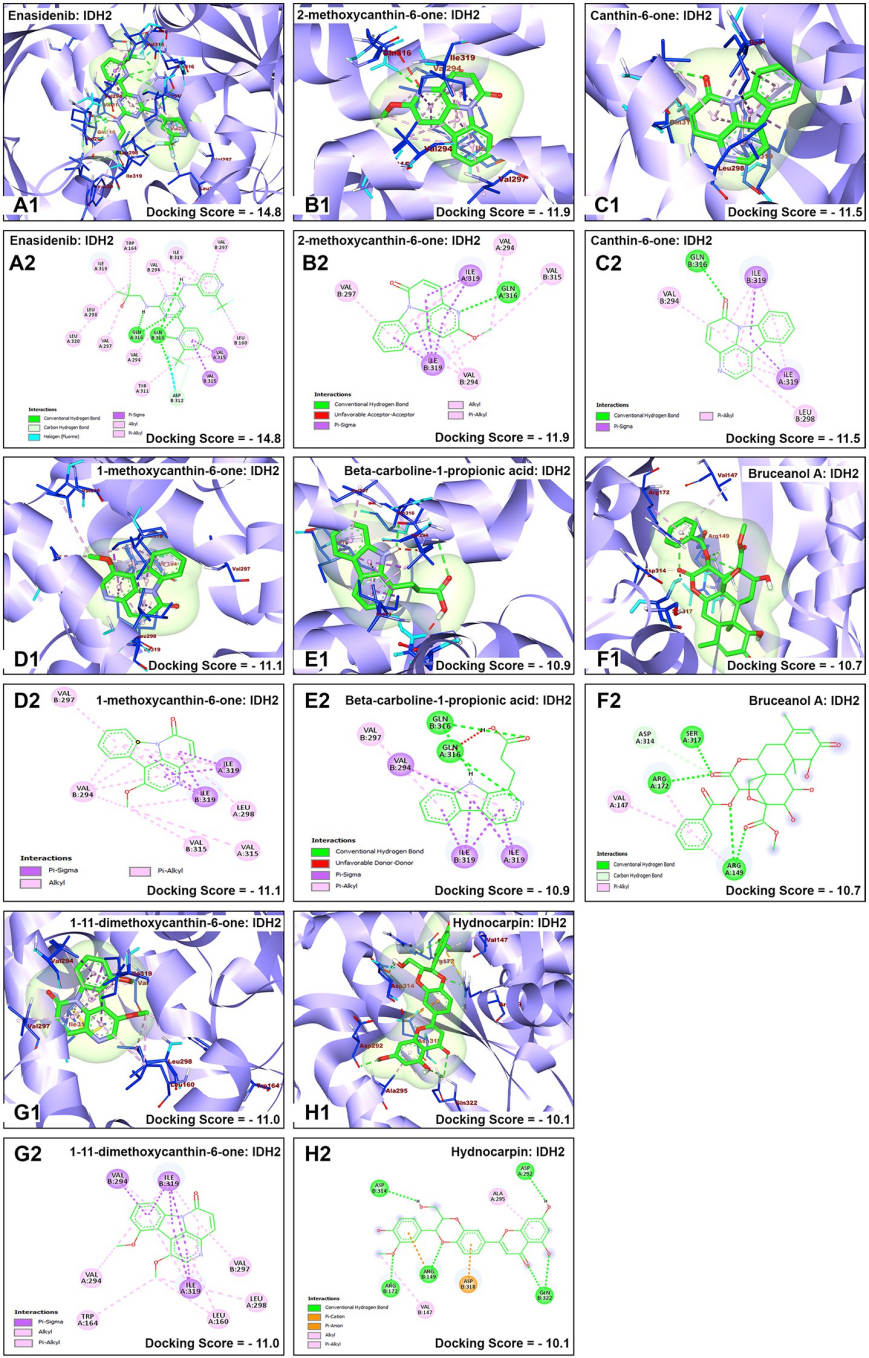
Molecular docking analysis of enasidenib and selected *B*. *antidysentrica* phytochemicals against IDH2 AML receptor. (1) 3D pose views of interaction of compounds with AML receptor IDH2. (2) 2D pose views of interaction of compounds with AML receptor IDH2.

**Fig 5:**
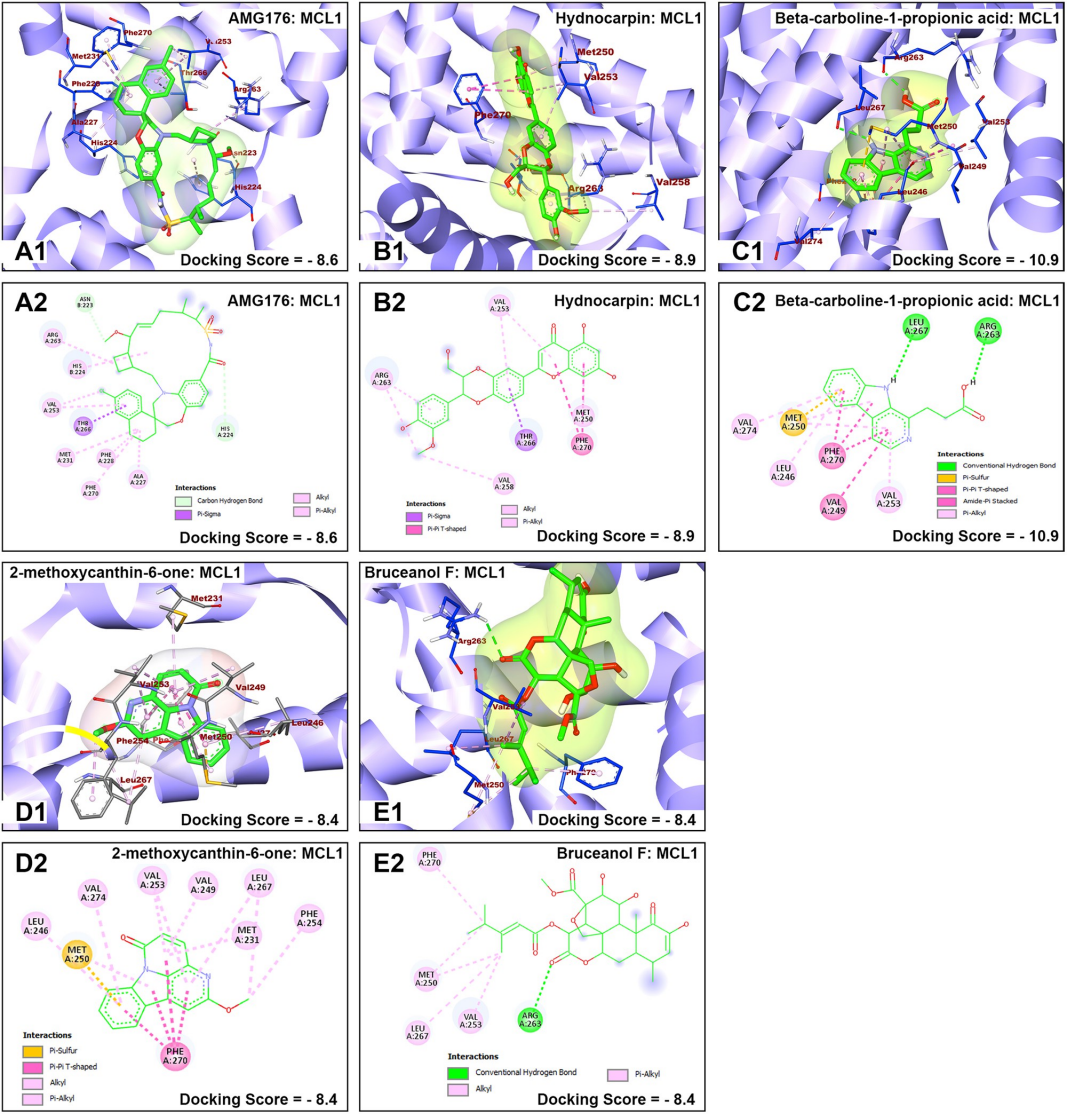
Molecular docking analysis of AMG176 and selected *B*. *antidysentrica* phytochemicals against MCL1 AML receptor. (1) 3D pose views of interaction of compounds with AML receptor MCL1. (2) 2D pose views of interaction of compounds with AML receptor MCL1.

**Fig 6:**
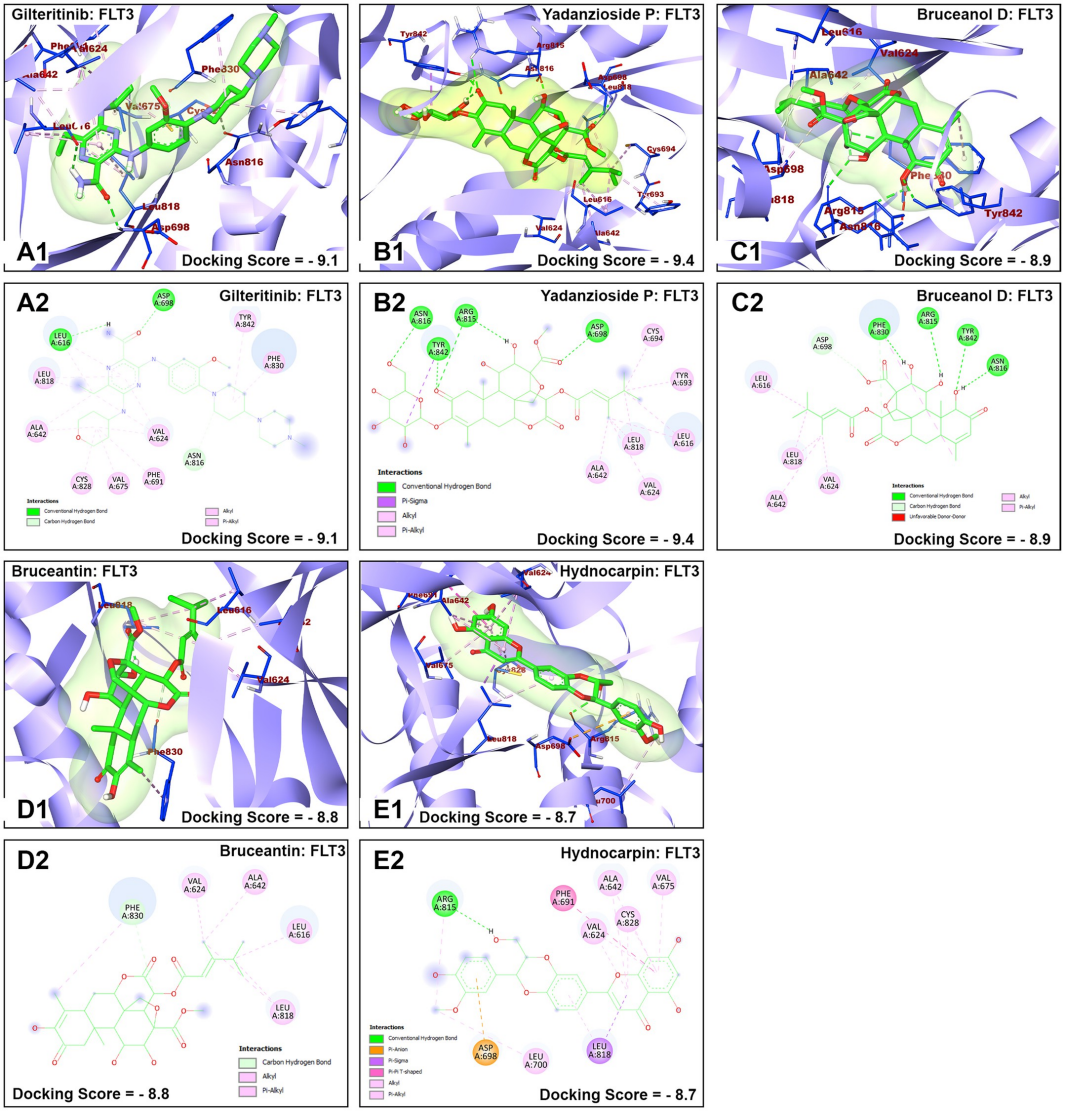
Molecular docking analysis of gilteritinib and selected *B*. *antidysentrica* phytochemicals against FLT3 AML receptor. (1) 3D pose views of interaction of compounds with AML receptor FLT3. (2) 2D pose views of interaction of compounds with AML receptor FLT3.

**Fig 7:**
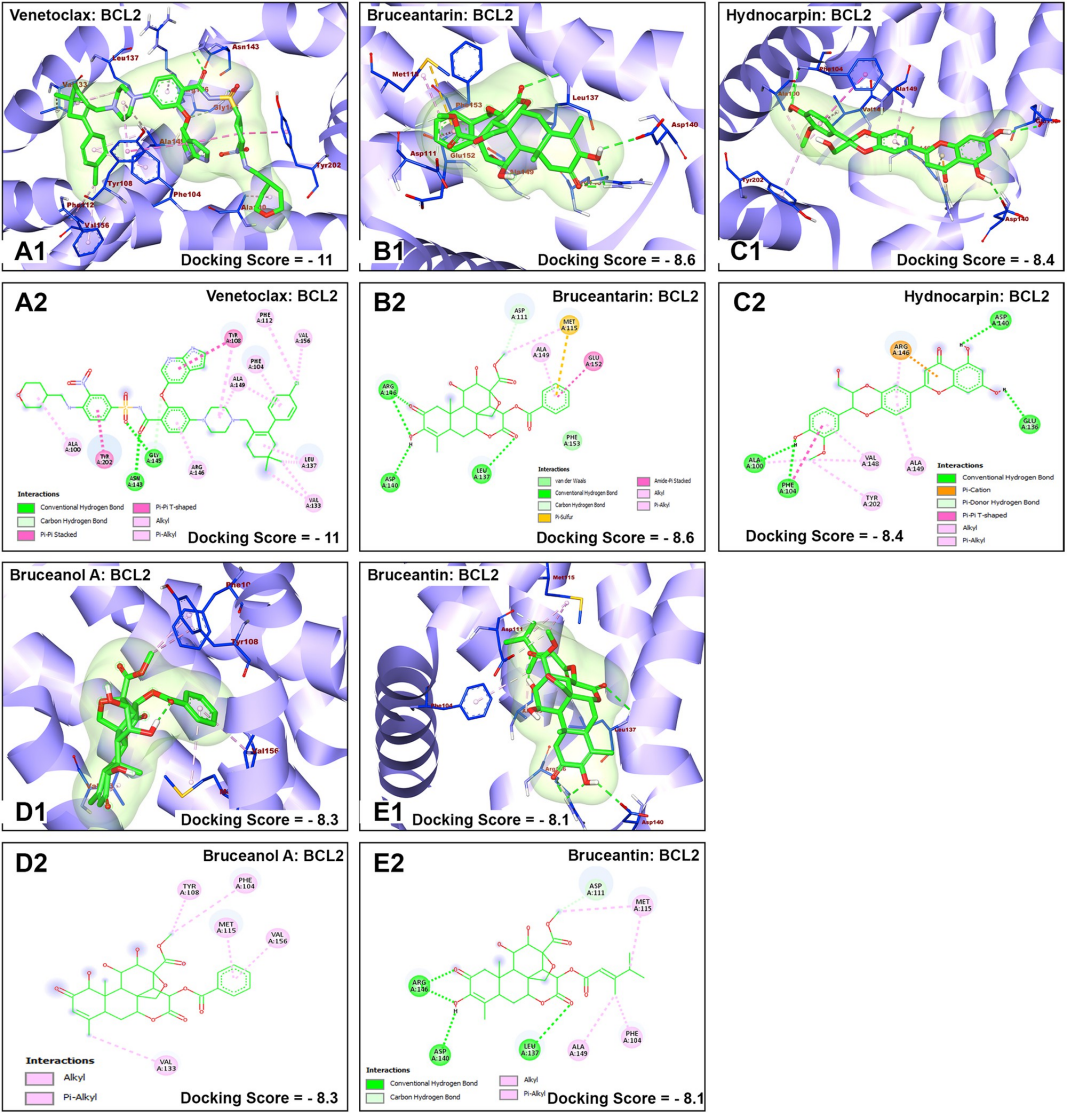
Molecular docking analysis of venetoclax and selected *B*. *antidysentrica* phytochemicals against BCL2 AML receptor. (1) 3D pose views of interaction of compounds with AML receptor FLT3. (2) 2D pose views of interaction of compounds with AML receptor BCL2.

The nature of interaction and interacting amino acid residues of the best docking poses in the binding sites of the receptors with selected phytochemicals was predicted using Protein-Ligand Interaction Profiler (PLIP) automated web tool and the results are summarized in in Table 1. The 3D and 2D view of the interactions between the selected *B*. *antidysentrica* phytochemicals and the AML receptors are shown in Figs 4–7.

**Table 1:** Binding affinity energies, nature of interaction and interacting amino acid residues between the compounds (small molecule drug and phytochemicals) and AML receptors.

Small molecule drug and phytochemicals	Target (PDB ID)	Docking score (kcal/mol)	p-value	Hydrophobic interaction	Hydrogen bonds
Enasidenib	IDH2 (5I96)	-14.8	1.21E-66	Leu160B, Trp164, Ile290, Val297, Val297B, Leu298, Trp306B, Val315	NA
2-methoxycanthin-6-one	IDH2 (5I96)	-11.9	9.44E-43	Trp164B, Val279B, Ile319	NA
Canthin-6-one	IDH2 (5I96)	-11.5	2.16E-38	Leu298, Ile319	NA
1-methoxycanthin-6-one	IDH2 (5I96)	-11.1	1.09E-33	Leu160, Leu298, Trp306B	Gln316
Beta-carboline-1-propionic acid	IDH2 (5I96)	-10.9	1.87E-32	Trp164B, Val279B, Ile319	NA
Bruceanol A	IDH2 (5I96)	-10.7	3.37E-31	Val147, Arg149, Arg172	Arg149, Arg172, Asp314
1,11-dimethoxycanthin-6-one	IDH2 (5I96)	-11.0	1.28E-28	Leu160, Trp164, Trp164B, Val297B	NA
Hydnocarpin	IDH2 (5I96)	-10.1	2.15E-20	NA	Arg149B, Lys299
AMG176	MCL1(6O6F)	-8.6	2.91E-13	Phe228, Met231, Val253, Thr266	NA
Hydnocarpin	MCL1(6O6F)	-8.9	1.85E-18	Met250, Val253, Phe254, Leu267, Phe270, His224B	NA
Beta-carboline-1-propionic acid	MCL1(6O6F)	-8.5	1.43E-11	Leu246, Phe270, Gly271, Leu290	Arg263
2-methoxycanthin-6-one	MCL1(6O6F)	-8.4	6.34E-10	Leu235, Leu246, Phe270, Leu290	NA
Bruceanol F	MCL1(6O6F	-8.4	1.73E-05	Met231, Phe270	His 224B, His224B, Arg263
Gilteritinib	FLT3 (6JQR)	-9.1	1.23E-16	Val675, Phe830	Leu616
Yadanzioside P	FLT3 (6JQR)	-9.4	2.53E-21	Leu616, Ala642, Tyr693, Asp698, Leu818, Phe830, Tyr842	Gln575, Leu616, Arg815
Bruceanol D	FLT3 (6JQR)	-8.9	1.62E-13	Leu616, Gly617, Gly619, Val624, Leu818, Phe830	Val624, Asp698, Phe830, Tyr842
Bruceantin	FLT3 (6JQR)	-8.8	5.54E-12	Leu616, Gly617, Gly619, Val624, Leu818, Phe830	Asp698, Phe830, Tyr842
Hydnocarpin	FLT3 (6JQR)	-8.7	1.75E-10	Val675, Phe691, Arg815, Leu818, Cys828, Phe830	Arg815
Venetoclax	BCL2 (6O0K)	-11	2.01E-52	Phe104, Tyr108, Val133, Leu137, Tyr202	NA
Bruceantarin	BCL2 (6O0K)	-8.6	2.24E-17	Phe104, Met115, Leu137, Lew137, Ala149, Phe153	Asp140
Hydnocarpin	BCL2 (6O0K)	-8.4	8.84E-14	Phe104, Arg146, Val148, Ala149	Arg146
Bruceanol A	BCL2 (6O0K)	-8.3	5.16E-12	Phe104, Met115, Val133, Glu152, Val156	NA
Bruceantin	BCL2 (6O0K)	-8.1	1.22E-08	Asp111, Met115, Leu137, Ala149, Phe153, Val156	Asp140

Most of the interactions between the target proteins and ligands (small molecule drugs and phytochemicals) (Table 1) are hydrophobic interactions. Analysis of marketed drugs revealed that the average number of hydrophobic atoms is 16 [70], indicating the importance of hydrophobic interactions in drug designing. Hydrophobic interactions increase the binding affinity between target-drug interfaces and enhance biological activity of complex molecules as well as help in stabilizing the biochemical environments of target-drug complexes [71]. Hydrophobic interactions are dominant for all receptors with both small molecule standard drugs and phytochemicals. However, for the receptor FLT3, there are also considerable formations of hydrogen bond interactions compared with the other receptors. Previous works done to understand the drug efficacy concluded that the binding affinity of the target-receptor molecules were increased by optimizing the hydrophobic interactions by captivating hydrogen bonding [71].

2-methoxycanthin-6-one showed interactions with receptor IDH2 forming hydrophobic interactions with Trp164B, Val279B, and Ile319 residues, with low binding energy of -11.9 kcal/mol next to the co-crystalized ligand pose with binding affinity score of −14.88 kcal/mol. Similarly, canthin-6-one, 1-methoxycanthin-6-one, beta-carboline-1-propionic acid, bruceanol A, 1,11-dimethoxycanthin-6-one showed consecutive binding affinity with mainly hydrophobic interaction indicating good binding mode with important interactions. Similar scenarios could be drawn for the remaining receptors. It is interesting to note that hydnocarpin showed more hydrophobic interactions and hydrogen bonds with receptor MCL1 and the binding energy is also lower compared to standard drug AMG176. This indicates that hydnocarpin can be considered a lead compound targeting MCL1 receptor. Similarly, Yadanzioside P showed more hydrophobic interactions and hydrogen bonds with lower binding energy to receptor FLT3 compared to standard drug gilteritinib, signifying its potential as a lead compound targeting FLT3.

In silico analysis should be complemented with other pharmacological profiling [72]. Compounds such as those that contain reactive groups, insoluble compounds, compounds that are too large/less extendable, or highly flexible compounds [66] should be screened.

Drug-likeness is related to molecular properties affecting pharmacodynamics and pharmacokinetics of the molecules. These molecular properties are related to some basic structural or physicochemical properties such as log *p* (partition coefficient), molecular weight (MW), Topological polar surface area (TPSA), hydrogen bond acceptors and donors count in a molecule. There are several sets of criteria to select ligands for a potential drug. Most notably, Lipinski [73] identified common properties that are frequently observed in approved compounds. They present a “rule of 5” for drug-like molecules (less than 5 hydrogen bond donors, less than 10 hydrogen bond acceptors, less than 500 dalton molecular weight, and less than 5 log *p* (partition coefficient between organic and aqueous phases). In addition, Veber’s rule [74] suggested that compounds which meet only the two criteria of 10 or fewer rotatable bonds and polar surface area equal to or less than 140 Å^2^ will have a high probability of good oral bioavailability in the rat. However, many of the most successful drugs do not fit these guidelines, and care should be taken in application of these guidelines [72].

Most of the investigated *B*. *antidysentrica* phytochemicals obeys most of the Lipinski’s and Veber’s rule similar to that of approved small molecule drugs as shown in Table 2. We used compounds with two or more Rule of 5 violations being used in drug development by medicinal chemists and industries [75] as a reference for drug-likeness evaluation of the phytochemicals in this work. Especially over the past two decades, the increasing significance of drugs not conforming to this rule is illustrated by the increase in molecular mass of approved oral drugs, compelling evidence to contradict the suggestion that molecular weight is a “drug-like” property [76, 77], Since natural products contain enormous scaffold diversity and structural complexity and typically possesses high molecular weights, a larger number of sp3 carbon atoms and oxygen atoms but fewer nitrogen and halogen atoms, higher numbers of H-bond acceptors and donors, lower calculated octanol–water partition coefficients (cLog *P* values, indicating higher hydrophilicity) and greater molecular rigidity compared with synthetic compound libraries, they are a main source of oral drugs ‘beyond Lipinski’s rule of five’ [76, 78], In addition, most of the compounds contain acceptable molecular weight and convenient TPSA values as shown in Table 3. In order to investigate majority of compounds for drug development purpose, compound with molecular weight of greater than 500 dalton and less than 3000 dalton are being used [78]. Therefore, the selected *B*. *antidysentrica* phytochemicals possesses sufficient drug-likeness and physicochemical properties suggesting their potential as therapeutic drug as shown in Tables 2 and 3.

**Table 2:** Lipinski’s rule of five and Veber’s rule for drug-likeness analysis of selected phytochemicals and small molecule drugs.

Compounds	Molecular weight (g/mol)	Lipophilicity (log p)	Hydrogen bond donors	Hydrogen bond acceptors	TPSA	ROTB	Number of Lipinski’s rule violations	Number of Veber’s rule violations
Phytochemicals								
1,11-dimethoxycanthin-6-one	280.28	2.12	0	4	107.59	2	0	0
1-methoxycanthin-6-one	250.25	2.39	0	3	43.6	1	0	0
2-methoxycanthin-6-one	250.25	2.73	0	3	43.6	1	0	0
Beta-carboline-1-propionic acid	240.26	2.11	2	3	65.98	3	0	0
Bruceanol A	542.53	0.7	3	11	165.89	5	2	1
Bruceanol D	548.58	1.25	3	11	165.89	6	2	1
Bruceanol F	548.58	2.28	3	11	165.89	6	2	1
Bruceantarin	542.53	1.11	3	11	165.89	5	2	1
Bruceantin	548.58	1.66	3	11	165.89	6	2	1
Canthin-6-one	220.23	2.42	0	2	34.37	0	0	0
Hydnocarpin	464.42	3.73	4	9	138.82	4	1	0
Yadanzioside P	710.72	-0.14	6	16	245.04	9	2	1
Small molecule drugs								
Enasidenib	473.4	3.5	3	14	109	8	2	0
AMG176	613.2	6.8	1	6	93.3	1	2	0
Gilteritinib	552.7	3.5	3	10	121	9	1	0
Venetoclax	868.4	8.2	3	11	183	14	3	2

TPSA: topological polar surface area, ROTB: number of rotatable bonds

**Table 3:** Calculated physicochemical properties and toxicity class of selected *B*. *antidysentrica* phytochemicals and small molecule drugs.

Compounds	Molecular weight (g/mol)	log *p*	Water solubility Silicos-IT class	GI absorption	BBB permeant	Abbott bioavailability score	Toxicity class
**Phytochemicals**							
1,11-dimethoxycanthin-6-one	280.28	2.12	Moderately soluble	High	No	0.55	4
1-methoxycanthin-6-one	250.25	2.39	Moderately soluble	High	yes	0.55	4
2-methoxycanthin-6-one	250.25	2.73	Moderately soluble	High	yes	0.55	3
Beta-carboline-1-propionic acid	240.26	2.11	Moderately soluble	High	yes	0.85	3
Bruceanol A	542.53	0.7	Soluble	Low	No	0.17	2
Bruceanol D	548.58	1.25	Soluble	Low	No	0.17	2
Bruceanol F	548.58	2.28	Soluble	Low	No	0.17	2
Bruceantarin	542.53	1.11	Soluble	Low	No	0.17	4
Bruceantin	548.58	1.66	Soluble	Low	No	0.17	2
Canthin-6-one	220.23	2.42	Moderately soluble	High	yes	0.55	4
Hydnocarpin	464.42	3.73	Poorly soluble	Low	No	0.55	5
Yadanzioside P	710.72	-0.14	Soluble	Low	No	0.17	2
**Small molecule drugs**							
Enasidenib	473.4	3.5	Poorly soluble	Low	No	0.55	4
AMG176	613.2	6.8	Poorly soluble	Low	No	0.55	4
Gilteritinib	552.7	3.5	Poorly soluble	High	No	0.17	4
Venetoclax	868.4	8.2	Insoluble	Low	No	0.17	4

**Toxicity class:** Class 1: fatal if swallowed (LD50 ≤ 5); Class 2: fatal if swallowed (5 < LD50 ≤ 50); Class 3: toxic if swallowed (50 < LD50 ≤ 300); Class 4: harmful if swallowed; (300 < LD50 ≤ 2000); Class 5: may be harmful if swallowed (2000 < LD50 ≤ 5000); Class 6: non-toxic (LD50 > 5000)

**Fig 8:**
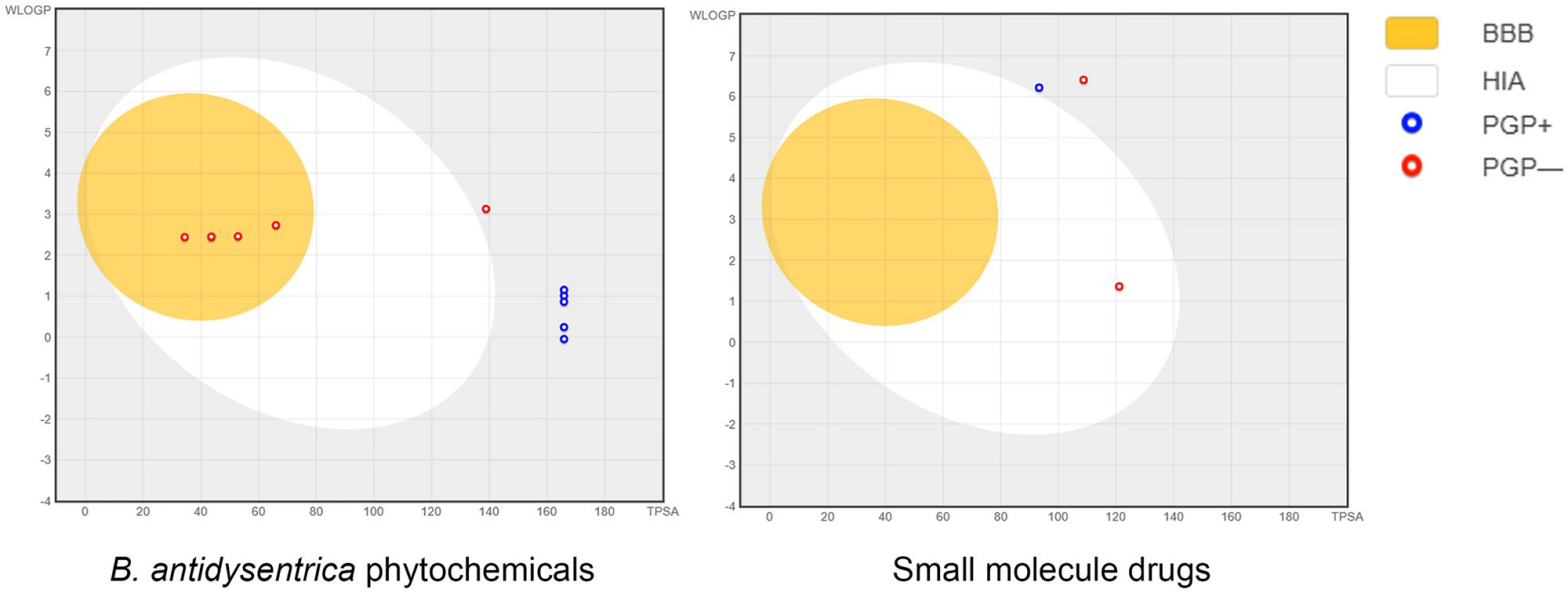
BOILED-Egg model of small molecule standard drugs and selected *B*. *antidysentrica* phytochemicals.

The predicted log *p* value and Abbott bioavailability score (greater than zero) revealed that the compounds have a substantial bioavailability and cross the cell membrane efficiently [78]. Gastrointestinal absorption (GIA) and blood–brain barrier (BBB) permeation of the phytochemicals are also comparable to that of standard small molecule approved drugs (Table 3). The phytochemicals exhibited acceptable gastrointestinal absorption which is an important property of a drug that is intended for mass treatment. In addition, phytochemicals 1-methoxycanthin-6-one, 2-methoxycanthin-6-one, beta-carboline-1-propionic acid and Canthin-6-one can cross the blood-brain barrier. The remaining compounds do not cross blood-brain barrier similar to that of small molecule approved drugs. The Brain Or Intestine EstimateD (BOILED-Egg) model prediction of GIA and BBB permeation for small molecule drugs and *B*. *antidysentrica* phytochemicals is presented in Fig 8. The compounds 2-methoxycanthin-6-one from phytochemicals and Venetoclax from the small molecule drugs were out of range of the BOILED-Egg model diagram.

Toxicity class of the phytochemicals range from class 2 (fatal) to 5 (may be harmful) and that of small molecule drugs are class 4 (harmful) which indicates that the phytochemicals have comparable to the acute toxicity to approved modern small molecule drugs.

Permeability glycoprotein (P-gp) is an ATP-binding cassette (ABC) membrane transporter, which limits cellular uptake, distribution, excretion and toxicity of compounds by acting as a unidirectional efflux pump to extrude its substrate from inside to outside of cells thereby obstructing cell internalization of chemotherapeutic agents [79, 80], From Table 4, it can be seen that 50% of the phytochemicals and small molecule drugs are not substrates of P-glycoprotein, demonstrating their desirable properties as potential chemotherapeutic agents. On the other hand, the remaining half of the phytochemicals and small molecule drugs are substrates of P-glycoprotein, suggesting that various strategies should be used to tackle this curb in the field of drug delivery and targeting.

**Table 4:** Interaction of selected *B*. *antidysentrica* phytochemicals and small molecule modern drugs with P-glycoprotein and cytochrome P450 isoenzymes.

Phytochemicals	Pgp substrate	CYP1A2 inhibitor	CYP2C19 inhibitor	CYP2C9 inhibitor	CYP2D6 inhibitor	CYP3A4 inhibitor
**Phytochemicals**						
1,11-dimethoxycanthin-6-one	No	Yes	No	Yes	Yes	Yes
1-methoxycanthin-6-one	No	Yes	No	No	No	Yes
2-methoxycanthin-6-one	No	Yes	No	No	No	No
Beta-carboline-1-propionic acid	No	Yes	No	No	No	No
Bruceanol A	Yes	No	No	No	No	No
Bruceanol D	Yes	No	No	No	No	No
Bruceanol F	Yes	No	No	No	No	No
Bruceantarin	Yes	No	No	No	No	No
Bruceantin	Yes	No	No	No	No	No
Canthin-6-one	No	Yes	No	No	No	No
Hydnocarpin	No	No	No	Yes	No	Yes
Yadanzioside P	Yes	No	No	No	No	No
**Small molecule drugs**						
Enasidenib	No	Yes	No	Yes	Yes	Yes
AMG176	Yes	No	No	No	No	No
Gilteritinib	No	No	Yes	No	No	No
Venetoclax	Yes	No	No	No	No	No

Cytochrome P450s (CYPs) represent a large class of heme-containing enzymes that catalyze the metabolism of multitudes of substrates including chemotherapeutic agents. Therefore, strategies to inhibit the enzymes are one approaches for the treatment and prevention of cancer [81]. The phytochemicals 1,11-dimethoxycanthin-6-one, 1-methoxycanthin-6-one, 2-methoxycanthin-6-one, beta-carboline-1-propionic acid, canthin-6-one inhibits CYP1A2 enzyme activity and that these inhibitory effects may contribute towards the cancer preventive action of the compounds. Similarly, phytochemicals 1,11-dimethoxycanthin-6-one, 1-methoxycanthin-6-one and hydnocarpin are inhibitors of CYP3A4 indicating their potential as chemotherapeutic agents.

## Conclusions

When a specific target gene or protein that is differentially expressed in cancer is found, the search for small molecules targeted therapies is usually performed using high throughput screening. Although this is the gold standard, it is a time consuming and expensive process. Therefore, computational drug discoveries have been extensively employed in assisting the process of finding lead compounds in such targeted therapies. In addition, starting this search from the empirical traditional knowledge of natural products in treating some ailments can therefore help in saving cost and time. Combining the computational approach with the empirical knowledge of natural products can even make the process faster and cheaper. Therefore, in this work, starting from empirical knowledge and using computational methods, we set out to find the compounds of *B*. *antidysentrica* involved in potentially treating AML. Based on the binding affinities as exhibited by the molecular docking studies and supported by the statistical analysis, the current study concludes that the 12 phytochemicals from *B*. *antidysentrica* can be considered as possible agents against IDH2, MCL1, FLT3 and BCL2 receptors. Hence, this study has partially recognized the traditionally claimed anti-leukemic activity of the plant. In addition, most of the phytochemicals were found to have acceptable drug-likeness and ADMET properties showing their potential as drug candidates. Similar in silico approaches can be applied the study of other traditional medicinal materials to identify new potential therapeutic compounds.

## Supporting information

S1 File 3D SDF of small molecule drugs used in this study. (RAR)

S2 File 3D SDF of all the investigated *B*. *antidysentrica* phytochemicals. (RAR)

S3 File 3D SDF of the random compounds used for statistical analysis of significance. (RAR)

S4 File 2D SDF of all investigated compounds. (RAR)

S5 File 2D PNG structures of all investigated compounds. (RAR)

S6 File Docking scores and p-values of small molecule drugs and phytochemicals. (XLSX)

## List of abbreviations

3D: three-dimensional
ADMET: absorption, distribution, metabolism, excretion, and toxicity
ADT: AutoDock Tools
AML: Acute myeloid leukemia (AML)
BBB: Blood–Brain Barrier
BCL2: B-cell lymphoma 2
FDA: Food and Drug Administration
FLT3: Fms-like Tyrosine Kinase 3
GIA: Gastrointestinal Absorption
IDH1: Isocitrate dehydrogenase
MCL1: Myeloid cell leukemia-1
PDB: Protein Data Bank
PLIP: Protein-Ligand Interaction Profiler
SDF: Structure Data File
SMILES: Simplified Molecular-Input Line-Entry System
TTD: Therapeutic Target Database
